# Reasons for allowing and refusing generic substitution and factors determining the choice of an interchangeable prescription medicine: a survey among pharmacy customers in Finland

**DOI:** 10.1186/s12913-020-4894-3

**Published:** 2020-02-03

**Authors:** Henriikka Nokelainen, Elina Lämsä, Riitta Ahonen, Johanna Timonen

**Affiliations:** School of Pharmacy / Social Pharmacy, Faculty of Health Sciences, Kuopio Campus, University of Eastern Finland, P.O.Box 1627, FI-70211 Kuopio, Finland

**Keywords:** Choice of medicine, Community pharmacy, Customer, Experience, Generic substitution, Interchangeable prescription medicine, Reference price system, Survey

## Abstract

**Background:**

Generic substitution (GS) was introduced in Finland in 2003 and supplemented with a reference price system (RPS) in 2009. Patients play a vital role in the acceptance of GS and the use of less expensive generic medicines. The objective of this study was to explore Finnish pharmacy customers’ experience with allowing and refusing GS. Specific aims were to investigate the reasons for (1) allowing and (2) refusing GS and (3) to determine the prescription medicine-related factors influencing the customer’s choice of an interchangeable prescription medicine.

**Methods:**

A questionnaire survey was conducted in February 2018. Questionnaires were handed out from 18 community pharmacies across Finland to customers ≥18 years who purchased for themselves a prescription medicine included in the RPS. A descriptive approach was used in the analysis using frequencies, the Chi-square test and Fisher’s exact test.

**Results:**

The final study material consisted of 1043 questionnaires (response rate 40.0%). Of the customers, 47.9% had both allowed and refused GS, 41.2% had only allowed GS and 6.0% had only refused GS. Customers had allowed GS because they wanted to lower their medicine expenses (75.5%), or because the prescribed medicine (30.8%) or medicine they had used before (27.4%) was unavailable at the pharmacy. The main reasons for refusing GS were an insignificant price difference between interchangeable medicines (63.3%) and satisfaction with the medicine used before (60.2%). The main factors influencing customers’ choice of an interchangeable prescription medicine were price (81.1%), familiarity (38.4%) and availability (32.8%). Customers who had allowed GS chose the medicine based on price. Customers who had only refused GS appreciated familiarity more than the price of the medicine.

**Conclusions:**

GS is a common practice in Finnish community pharmacies. The price of the medicine was the most important factor affecting customers’ decision to allow or refuse GS and the choice of an interchangeable prescription medicine. Thus, customers should receive information about medicine prices at the pharmacy in order to help them make their decision. However, individual needs should also be taken into account in counselling because customers regard several factors as important in their choice of an interchangeable medicine.

## Background

Generic medicines (GM) provide patients with medical treatment that is as effective as brand name products but less expensive [1]. During the last few decades, generic substitution (GS) has become important in promoting the use of less expensive GM. In Europe, GS is in use in over 30 countries [2]. However, there are differences in practices between countries, because GS can be permissive or mandatory, the former being slightly more common. Nevertheless, a physician or a patient can prohibit or decline substitution. GS has been supplemented with a reference price system (RPS) in almost 30 European countries. These systems aim to reduce medicine costs by encouraging the use of less expensive medicines and to increase price competition between pharmaceutical companies [3, 4].

Patients play a vital role in the acceptance of GS and the use of less expensive medicines. Patients’ attitudes towards GS have been mainly positive [5–7] although some patients are reluctant to substitute their medicines [6, 8, 9]. Refusing GS causes extra costs for both patients and society [10]. Therefore, it is important to understand why patients allow or refuse GS. Many studies have reported that patients allow or would be willing to allow GS based on a physician’s [8, 11–13] or pharmacist’s recommendation [5, 7, 8, 11, 12] and to lower their medicine expenses [5, 7, 8, 11, 14]. Other studies report that patients are reluctant to substitute their medicine because they value a familiar product they have used before or a brand name product they trust [6, 11, 14, 15]. It has also been found that negative perceptions regarding the effectiveness and safety of GM make patients less willing to accept a substitute [11, 15–17].

The introduction of GS changes patients’ role since they can make the final decision about the prescription medicinal product they choose from generic alternatives at the pharmacy. Consequently, it is important to know which prescription medicine-related factors affect patients’ choices in order to develop and provide information for patients to support their decision making. Although a large number of studies have examined GS from different perspectives, only a few have explored prescription medicine-related factors that affect patients’ choice of prescription medicine [8, 9, 18–20].

In Finland, GS was introduced by law in 2003 [21]. The law obliges pharmacists to offer customers the opportunity to substitute their prescription medicines with the cheapest or close to the cheapest interchangeable medicine unless the prescriber prohibits the substitution. The customer can also refuse the substitution. Pharmacists are also required to inform customers about medicine prices and other factors affecting their choice of medicinal product [21]. Since 2016, price counselling has had to include information about the least expensive interchangeable medicine at the point of dispensing [22].

The Finnish Medicines Agency Fimea maintains a list of interchangeable medicines [23]. Interchangeable medicines contain the same quantity of the same active substance, have the same pharmaceutical form (with the exception that tablets and capsules are regarded as interchangeable) and are biologically equivalent; the medicines’ safety profiles and therapeutic indexes are also sufficiently broad to permit substitution. At the beginning of 2019, the list of interchangeable medicines consisted of 5429 products, which is about 59% of all human medicinal products with a marketing authorisation in Finland (*n* = 9198) [24].

In 2009, GS was supplemented with a RPS in which interchangeable medicines are clustered into reference price groups, for each of which a reference price is defined [25, 26]. The reference price sets limits on the price for which reimbursement is available and is determined by adding €0.50 to the cheapest interchangeable medicine’s retail price [26]. This €0.50 price difference is called the reference price band. If a customer decides to purchase a prescribed medicine priced higher than the reference price, the reimbursement is calculated from the reference price, and the customer pays the excess in addition to the co-payment [27]. Reference price groups, medicinal products included in the groups and reference prices are determined quarterly [26]. However, pharmaceutical companies may update the prices of their products twice a month, so the products included in the price band may vary during each quarter [28].

In Finland, patients’ experience with allowing and refusing GS and the factors determining their choice of prescription medicine were investigated in the early stage of GS [5–7, 20]. Since that time, many legislative reforms have been introduced to promote the use of interchangeable medicines and price competition between pharmaceutical companies, such as the introduction of a RPS in 2009 [25] and greater price counselling at pharmacies in 2016 [22]. The aim of this study was to investigate Finnish pharmacy customers’ experience with allowing and refusing GS 15 years after the implementation of GS and after the legislative changes. The specific aims were to investigate the reasons why Finnish pharmacy customers (1) allow or (2) refuse GS and (3) which medicine-related factors affect their decision when choosing an interchangeable prescription medicine.

## Methods

A questionnaire survey was carried out in February 2018. The survey was targeted at pharmacy customers aged ≥18 who were purchasing for themselves a prescription medicine included in the RPS. Questionnaires were distributed from 18 different-sized community pharmacies recruited from all six Regional State Administrative Agencies’ areas in mainland Finland: Southern Finland, Southwestern Finland, Western and Central Finland, Eastern Finland, Northern Finland, and Lapland [29]. One university pharmacy branch (owned by a university, but operating like a privately-owned pharmacy), one privately-owned big city pharmacy and one privately-owned small rural pharmacy were recruited from each area using convenience sampling. The number of questionnaires delivered to each pharmacy was adapted according to the number of prescriptions dispensed daily at the pharmacy and varied between 30 and 300. Pharmacists were informed about the study in writing. They were requested to tell all eligible customers about the study after the dispensing situation and offer them a questionnaire. Customers filled in the questionnaire at home and mailed it to the researchers. The questionnaires were distributed as long as there were forms left, but for a maximum of 2 weeks. Pharmacists were not required to keep a list of customers who declined to participate. After the study period, the pharmacies reported the number of remaining questionnaires in order to compute the response rate. Altogether, 2606 questionnaires were distributed. Reminders could not be sent because customers were recruited anonymously. A similar method has been successfully used in a few earlier studies targeted at Finnish pharmacy customers [7, 30, 31].

The four-page questionnaire consisted of 21 questions (see Additional file 1). The questionnaire was created for this study and based on earlier Finnish studies [5–7, 20, 31] and on national legislative requirements set for the implementation of GS and RPS [32–34]. The questionnaire was initially tested for face validity by five faculty members with experience of the design of questionnaire surveys. Thereafter, the pilot test was conducted in a local pharmacy. The pilot respondents were interviewed after filling in the questionnaires to check the content validity. Minor revisions were made as a result.

This paper reports the results from five questions regarding patients’ decisions to allow or refuse GS, the reasons, and the factors influencing their choice of an interchangeable prescription medicine. The questions regarding the reasons for allowing or refusing GS and the factors influencing the choice of an interchangeable prescription medicine were based on earlier surveys to Finnish medicine users about GS with minor revisions [5–7, 20]. Respondents’ experience with GS was explored by using two structured questions: “Have you ever substituted your prescription medicine with an equivalent medicinal product at the pharmacy?” and “Have you ever chosen not to substitute your prescription medicine with a cheaper equivalent medicinal product offered by the dispenser?”. Respondents were instructed to reply “Yes” or “No”. Respondents who had allowed or refused GS were asked to specify their reasons using a list of several fixed responses to choose from, and there was also space for a freely worded answer. The factors affecting respondents’ choice of prescription medicine were explored with the question: “If your physician has prescribed you a medicine which you can substitute with another, equivalent medicinal product at the pharmacy, which of the following factors matter when you are choosing a prescription medicine at the pharmacy?”. The question had a list of several fixed responses to choose from and also space for a freely worded answer. Each respondent’s gender, area of residence, education and current use of prescription medicines were obtained using structured questions and the year of birth using an open-ended question.

The data was analysed using the Statistical Package for the Social Sciences software (IBM SPSS Statistics for Windows, Version 25.0. Armonk, NY: IBM Corp). A descriptive approach was used in the analysis using frequencies, percentages and cross-tabulations. The Chi-square test and Fisher’s exact test were used to analyse associations between answers and background factors. Statistical significance was determined as *p*-value < 0.05. In the analysis respondents’ ages were placed into one of four groups (18–34, 35–59, 60–74 and 75 years or older).

The study setting and research process complied with local and national ethical instructions for research; this study did not require ethical approval [35, 36].

## Results

Altogether 1045 questionnaires were returned, two of which were blank. Consequently, the final study material consisted of 1043 questionnaires, giving a response rate of 40%. Most of the respondents were female (70.5%) and 60–74 years old (45.3%) (Table 1). The respondents’ ages ranged from 18 to 95 years, mean 62.2 (SD 14.9) and median 65 (IQR 16.0) years. There were respondents from all six areas of Finland.

**Table 1:** Characteristics of the study respondents (*N*=1043)

	n	%
Gender (*n*=1039^a^)		
Male	307	29.5
Female	732	70.5
Age in years (*n*=1007^a^)		
18–34	75	7.4
35–59	280	27.8
60–74	456	45.3
≥ 75	196	19.5
Area of residence (*n*=1030^a^)		
Southern Finland	184	17.9
Southwestern Finland	78	7.6
Western and Central Finland	251	24.4
Eastern Finland	192	18.6
Northern Finland	242	23.5
Lapland	83	8.1
Education (*n*=1027^a^)		
Basic education qualification	226	22.0
Vocational upper secondary qualification or vocational college diploma	397	38.7
Matriculation examination	117	11.4
Lower university degree	144	14.0
Higher university degree	143	13.9
Current use of prescription medicines (*n*=1023^a^)		
Regularly	606	59.2
Temporarily	102	10.0
Both regularly and temporarily	315	30.8

^a^Some of the respondents did not report their gender, age, area of residence, education or use of prescription medicines

### Respondents’ experience with GS

Of the respondents, 47.9% had both allowed and refused GS, 41.2% had only allowed GS, and 6% had only refused GS (Table 2). A small number of respondents (4.9%) had no experience with GS. Respondents who had no experience with GS were more likely to be aged 18–34 years than those who had experienced GS (*p* = 0.033). Respondents who had only refused GS were more likely to be from Lapland than those who had allowed or had no experience with GS (*p* = 0.035). Respondents who had both allowed and refused GS were less likely to have a basic education qualification than those who had only allowed or only refused GS or had no experience (*p* = 0.012). Instead, they had more often completed vocational education (*p* = 0.023). They were also less likely to use prescription medicines regularly compared to respondents who had only allowed or only refused GS or had no experience (*p* = 0.038). Respondents who had only refused GS or had no experience with it were less likely to use prescription medicines both regularly and temporarily than respondents who had allowed GS (*p* = 0.004).

**Table 2:** Differences in characteristics as regards respondents’ experience with GS (*N*=1043)

Characteristic	Respondents who had only allowed GS		Respondents who had only refused GS		Respondents who had both allowed and refused GS		Respondents who had no experience with GS		*p*-value^a^
	n	%	n	%	n	%	n	%	
All (*n*=1020^b^)	420	41.2	61	6.0	489	47.9	50	4.9	
Gender (*n*=1016^c^)									
Male	114	27.3	21	34.4	142	29.1	20	40.0	0.228
Female	303	72.7	40	65.6	346	70.9	30	60.0	0.228
Age in years (n=984^c^)									
18–34	39	9.5	3	5.4	26	5.5	7	14.6	**0.033**
35–59	108	26.3	13	23.2	148	31.6	10	20.8	0.151
60–74	189	46.0	31	55.4	208	44.3	18	37.5	0.294
≥ 75	75	18.2	9	16.1	87	18.6	13	27.1	0.469
Area of residence (*n*=1007^c^)									
Southern Finland	82	19.9	5	8.5	88	18.1	6	12.0	0.121
Southwestern Finland	38	9.2	1	1.7	35	7.2	3	6.0	0.200
Western and Central Finland	98	23.7	17	28.8	112	23.1	19	38.0	0.103
Eastern Finland	69	16.7	11	18.6	99	20.4	9	18.0	0.566
Northern Finland	98	23.7	14	23.7	112	23.1	10	20.0	0.948
Lapland	28	6.8	11	18.6	39	8.0	3	6.0	**0.035**
Education (*n*=1005^c^)									
Basic education qualification	98	23.7	16	26.2	82	17.0	16	32.0	**0.012**
Vocational upper secondary qualification or vocational diploma	146	35.4	18	29.5	210	43.7	17	34.0	**0.023**
Matriculation examination	54	13.1	8	13.1	48	10.0	6	12.0	0.520
Lower university degree	67	16.2	7	11.5	64	13.3	6	12.0	0.524
Higher university degree	48	11.6	12	19.7	77	16.0	5	10.0	0.122
Current use of prescription medicines (*n*=1000^c^)									
Regularly	247	60.2	40	66.7	273	56.6	32	66.7	**0.038**
Temporarily	36	8.8	10	16.7	47	9.8	9	18.8	0.056
Both regularly and temporarily	127	31.0	10	16.7	162	33.6	7	14.6	**0.004**

^a^Chi-square test and Fisher’s exact test; statistically significant *p*-values (*p* < 0.05) are in bold. ^b^Some of the respondents did not answer the questions concerning their experience with allowing or refusing GS. ^c^Some of the respondents did not report their gender, age, area of residence, education or use of prescription medicines

### Reasons for allowing and refusing GS

The main reason for allowing GS was the respondent’s desire to lower medicine expenses (75.5%) (Table 3). The other common reasons were situations when the prescribed medicine (30.8%) or the medicine the respondent had previously used (27.4%) was unavailable at the pharmacy.

**Table 3:** Reasons for allowing and refusing GS

	n	%^a^
Reasons for allowing GS (*n*=864^b^)		
Willingness to lower medicine expenses	652	75.5
The prescribed medicine was unavailable at the pharmacy	266	30.8
The previously used medicine was unavailable at the pharmacy	237	27.4
Pharmacist’s recommendation	225	26.0
Dissatisfaction with the medicine that was used before	31	3.6
Physician’s recommendation	23	2.7
Some other reason^c^	39	4.5
Reasons for refusing GS (*n*=528^d^)		
Too small a difference in price of the interchangeable medicines	334	63.3
Satisfaction with the medicine used before	318	60.2
The interchangeable medicine that the customer wanted was unavailable at the pharmacy	72	13.6
Customer’s concern that the interchangeable medicine will be less effective than the medicine used before	67	12.7
Choice of interchangeable medicine was made during physician visit	59	11.2
Too many switches before a suitable one was found	48	9.1
Customer’s concern that the interchangeable medicine will be less effective than the one prescribed by the physician	42	8.0
Customer’s medicines are subject to special reimbursement^e^, so the customer would not have benefited from the saving	26	4.9
Customer did not receive enough information about different alternative products	22	4.2
Fear of mixing up medicines	15	2.8
Some other reason^f^	72	13.6

^a^Respondents may have chosen several options

^b^Only customers who had experience with allowing GS answered this question. However, some did not report reasons for allowing GS

^c^E.g. willingness to purchase a Finnish product, the medicine was substituted in order to receive full reimbursement, and the previously used medicine was no longer manufactured

^d^Only customers who had experience with refusing GS answered this question. However, some did not report reasons for refusing GS

^e^In Finland, reimbursements for medicines are divided into three rates: basic reimbursement (40% of the prescription medicine price), lower special reimbursement (65%) and higher special reimbursement (100%, the customer pays a €4.50 co-payment per prescription medicine purchase) [37]

^f^E.g. the cost of the medicine is not paid by the customer, willingness to purchase a Finnish product, handy packet, and customer’s experience or fear of side effects

The most common reasons for refusing GS were too small a difference in price between interchangeable medicines (63.3%) and the fact that the medicine had been used before and was considered good (60.2%) (Table 3).

### Factors determining the choice of interchangeable prescription medicine

The three most important factors affecting respondents’ choice of interchangeable prescription medicine were price (81.1%), familiarity (38.4%) and availability of the medicine (32.8%) (Table 4). Respondents who had allowed GS and those who had no experience with GS were more likely to choose the medicine on the basis of price than those who had only refused GS (*p* < 0.001). Furthermore, respondents who had allowed GS and those who had no experience with GS were more likely to choose the medicine based on its availability than those who had only refused GS (*p* = 0.032). In contrast, for respondents who had only refused GS, familiarity with the medicine was the most important factor in their choice of interchangeable prescription medicine compared to other respondents (*p* < 0.001). Furthermore, respondents who had refused GS valued the fact that the medicine was Finnish more often than other respondents (*p* = 0.014).

**Table 4:** Factors that matter when respondents choose an interchangeable prescription medicine (*N*=1043)

	All (*n*=1030^a^)		Respondents who had only allowed GS (*n*=417^a,b^)		Respondents who had only refused GS (*n*=60^a,b^)		Respondents who had both allowed and efused GS (*n*=486^a,b^)		Respondents who had no experience with GS (*n*=47^a,b^)		*p*-value^d^
	n	%^c^	n	%^c^	n	%^c^	n	%^c^	n	%^c^	
Price of the medicine	835	81.1	374	89.7	28	46.7	386	79.4	33	70.2	**< 0.001**
Familiarity with the medicine	396	38.4	108	25.9	41	68.3	221	45.5	19	40.4	**< 0.001**
Availability of the medicine	338	32.8	135	32.4	11	18.3	174	35.8	12	25.5	**0.032**
The medicine is Finnish	198	19.2	63	15.1	14	23.3	111	22.8	6	12.8	**0.014**
Opportunity to halve the tablet	190	18.4	74	17.7	14	23.3	93	19.1	6	12.8	0.524
Medicine pack	166	16.1	60	14.4	6	10.0	89	18.3	6	12.8	0.193
Excipients contained in the product	131	12.7	32	7.7	4	6.7	90	18.5	3	6.4	**<0.001**
Manufacturer of the medicine / pharmaceutical company	59	5.7	12	2.9	11	18.3	35	7.2	1	2.1	**<0.001**
Tablet/capsule shape	59	5.7	17	4.1	4	6.7	37	7.6	1	2.1	0.097
Product name / brand / name of the medicine	14	1.4	1	0.2	3	5.0	10	2.1	0	0.0	**0.007**
Tablet/capsule colour	11	1.1	3	0.7	0	0.0	7	1.4	1	2.1	0.438
Other reason^e^	35	3.4	10	2.4	5	8.3	16	3.3	4	8.5	**0.025**

^a^Some of the respondents did not answer the question concerning the factors that affected their choice of an interchangeable prescription medicine. ^b^Some of the respondents did not answer the questions concerning their experience with GS. ^c^The respondents may have chosen several options. ^d^Chi-square test and Fisher’s exact test; statistically significant *p*-values (*p* < 0.05) are in bold. ^e^E.g. recommendation or opinion of the physician or pharmacist

## Discussion

In this study, a large majority of Finnish pharmacy customers had experienced GS. Almost 90% had substituted their medicines at least once, and nearly half of those were customers who had only allowed GS. The results indicate that GS is a common practice in Finnish community pharmacies. According to studies conducted in the early stage of GS in Finland, GS represented a significant pharmaceutical policy reform and had been implemented effectively in practice [38]. Finns look favourably on the opportunity to substitute their medicines and they consider less expensive GM effective and safe [5, 7]. Positive attitudes towards and experience with GS and less expensive medicines have been shown to increase people’s willingness to substitute their medicines [9, 11, 12]. In addition, according to figures published by the Social Insurance Institution of Finland and Finnish statistics on medicines, the number of substitutions has increased and the number of refusals has decreased since 2009 when the RPS was introduced [39, 40].

In the present study, a minority of customers had no experience with GS, although the questionnaires were distributed to customers who were purchasing for themselves a prescription medicine included in the RPS. There may be several reasons for this. First, their prescribed medicine had always been the cheapest interchangeable medicine at the point of dispensing. Second, pharmacists did not tell them about the cheaper alternatives and thus did not offer them the opportunity to substitute their prescription medicine. Third, the customer would have been unaware of the substitution if the pharmacist substituted the medicine without asking the customer.

According to this study, there were few differences between pharmacy customers’ characteristics and their experience with allowing or refusing GS. Differences mainly occurred among those who had both allowed and refused GS. Interestingly, pharmacy customers from Lapland were more likely to refuse GS than customers from other areas. This may result from the fact that there are differences in morbidity between Lapland and other regions of Finland [41], and some patient groups may refuse GS more often than others [42, 43]. However, we did not ask customers to specify their medications in this survey and we are therefore unaware of what medicines they refused to substitute. Further studies on this are needed in the future. It is also possible that the result is biased by the small number of respondents from Lapland. In contrast to some previous studies, gender was not associated with GS experience in our study [12, 44].

In this study, GS was mainly allowed by pharmacy customers because they wanted to lower their medicine expenses, which is in line with earlier studies conducted in Finland [5, 7] and other countries [8, 11, 14, 44]. The other main reasons for allowing GS were related to the unavailability of the prescribed medicine, which was an interesting finding. The current study showed that the unavailability of a prescribed medicine has become a more common reason to change to another interchangeable medicine (30.8%) compared to a Finnish population survey conducted 10 years earlier in which this reason was reported by 19.6% of participants [5]. This study did not reveal the reasons behind this finding, but possible reasons can be discussed. Nowadays, the number of medicinal products eligible for substitution is large [24] and it is impossible for pharmacies to stock all alternatives. A prescription medicine could also have been unavailable because it was in short supply. A previous study has reported that medicine shortages are common in community pharmacies in Finland because the large majority of pharmacies suffer from medicine shortages daily or almost daily [45]. According to figures published by the Finnish Medicines Agency, the number of medicine shortages reported by marketing authorization holders has tripled in Finland since 2012 [46]. However, medicine shortages seldom cause problems at pharmacies because GS enables pharmacists to substitute the customer’s medicine with an available interchangeable product [45]. Nevertheless, this finding would be an interesting topic to study in the future.

According to this study, the main reasons for refusals were too small a price difference between interchangeable medicines and satisfaction with the medicine used before. Our results differ from previous Finnish studies in that nowadays an insignificant price margin is a more common reason for refusing GS than in the early stages of GS (63.3% versus 19 and 35%) [6, 7]. In Finland, the price band has been narrowed twice from €3 to €0.5 during 2009–2017 [4, 47]. Consequently, it is possible that the €0.50 price band is already so narrow that it is an insufficient incentive for some customers to substitute their medicine.

In contrast to earlier Finnish studies [5, 7], our results indicate that customers are not as dependent on the pharmacist’s or physician’s opinion in their decision to allow or refuse GS than in the early stage of GS. In many earlier studies, the pharmacist’s or physician’s recommendations were among the main reasons why customers allowed GS or were more willing to accept GS in the future [5, 7, 8, 11–13]. In Finland, at the beginning of the GS reform [6, 7], patients wanted to ask their physician’s opinion before they allowed GS or they had already chosen the product at the physician’s office and then refused the substitution at the pharmacy [6]. It now seems that GS practice is familiar to customers and they can make their decisions by themselves.

This study found that a large majority of pharmacy customers choose their interchangeable prescription medicine based on price. The same finding emerged from a survey conducted 10 years earlier in Finland [20]. However, there were some differences in the factors affecting the choice of medicine in terms of pharmacy customers’ experience with GS. Customers who had allowed GS and customers who had no experience with GS considered price the most important factor in their choice of medicine. In contrast, customers who had only refused GS considered familiarity more important than price in their choice of medicine. Nonetheless, our results highlight the importance of price information in GS situations at pharmacies as a way of supporting customers’ decision making. In addition, because pharmacy customers appreciate different factors in their choice of interchangeable medicine, pharmacies should also give information about such factors. In Finland, pharmacists are required by law to inform customers not just about medicine prices but also about other factors affecting the choice of medicinal product [21, 22]. In the future, it would be interesting to study how patient counselling related to interchangeable medicines and GS is practised in pharmacies and to determine whether counselling meets the legal requirements. This should be studied from the perspective of pharmacists and pharmacy customers.

### Strengths and limitations

This study has some strengths and limitations. The study method was suitable for reaching the target group, i.e. customers who had experience with GS because questionnaires were distributed to customers who were purchasing for themselves prescription medicines included in the RPS. The study sample was also large, involving pharmacy customers across mainland Finland. The response rate of 40% is typical for this kind of method where it was impossible to send reminders [7, 29, 30]. There are no comparable statistics on Finnish pharmacy customers’ characteristics. Pharmacists were instructed to offer the questionnaire to all eligible customers, but they still may have used convenience sampling. Because customers were recruited anonymously, no information was gained about the characteristics of the customers to whom the questionnaire was offered but who declined to participate or who did not return the questionnaire. Compared to the statistics of Finnish population groups who have received reimbursement for medicine costs under the Health Insurance Scheme [48], men and customers aged 18–34 and 60–74 were under-represented in the present study. This may result from the fact that women and older people tend to respond to surveys more than men and younger adults [49, 50]. However, our study respondents’ current use of prescription medicines, gender and age distributions were similar to those of earlier Finnish studies conducted among pharmacy customers with similar methods [7, 31]. Accordingly, we suggest that the findings of this study can be generalized, with caution, to customers purchasing their prescription medicines at Finnish community pharmacies.

The majority of the questions reported in this paper were based on earlier Finnish population surveys [5, 6, 20] and a survey of Finnish pharmacy customers [7]. The questionnaire was tested for face validity and content validity before the survey. Furthermore, the separate response rates for the questions reported in this paper were 95–99%, illustrating that the respondents found the questions understandable.

## Conclusions

GS is a common practice in Finnish community pharmacies. A large majority of pharmacy customers have experience with allowing GS and half of customers have experience with refusing it. Pharmacy customers allow GS because they want to lower their medicine expenses. However, when the price difference between interchangeable medicines is too small, customers more frequently refuse GS. Also, some customers want to use familiar medicines and therefore refuse substitution. The most important factor affecting the customer’s choice of interchangeable medicine is price. It is therefore important that customers receive information about medicine prices at the pharmacy in order to support their decision making in the GS situation. However, individual needs should also be taken into account in counselling because customers appreciate several other factors in their choice of an interchangeable medicine.

## Supplementary information

**Additional file 1.** The questionnaire used in the current study. Survey for pharmacy customers regarding generic substitution and selection of prescription medicines

## Data Availability

The datasets used and analysed during the current study are not publicly available, but are available from the corresponding author upon reasonable request.

## Abbreviations

GM: Generic medicines
GS: Generic substitution
RPS: Reference price system
