# Supplementary material for: Reasons for allowing and refusing generic substitution and factors determining the choice of an interchangeable prescription medicine: a survey among pharmacy customers in Finland
Source: BMC Health Serv Res. 2020 Feb 3;20:82. doi: 10.1186/s12913-020-4894-3 (PMC6998302; doi:10.1186/s12913-020-4894-3)
Supplement: Supplementary file 1 — Additional file 1. The questionnaire used in the current study. Survey for pharmacy customers regarding generic substitution and selection of prescription medicines [file 12913_2020_4894_MOESM1_ESM.pdf]

University of Eastern Finland
Faculty of Health Sciences
School of Pharmacy
February 2018

UNIVERSITY OF EASTERN FINLAND

Additional file 1: Survey for pharmacy customers regarding generic substitution and selection of prescription medicines

Answer the questions by circling the number of the appropriate response option. If necessary, write the answer in the space reserved. It is important to answer all the questions.

1. Your gender?

1 Male
2 Female

2. Your year of birth? ___________________

3. Area where you live?

1 Southern Finland
2 Southwestern Finland
3 Western and Inland Finland
4 Eastern Finland
5 Northern Finland
6 Lapland

4. Your education?

1 Basic education qualification (comprehensive school, middle school and primary school)
2 Vocational upper secondary qualification or vocational college diploma
3 Matriculation examination
4 Lower university degree
5 Higher university degree

5. Are you currently using

1 Prescription medication regularly (e.g. medicines for high blood pressure)
2 Prescription medication only temporarily (e.g. antibiotics, painkillers taken as needed)
3 Both

THE FOLLOWING QUESTIONS CONCERN YOUR VISIT TO SEE A PHYSICIAN

6. Has your physician told you it is possible to substitute the medicine he/she has prescribed with a cheaper medicinal product at the pharmacy?

1 Yes
2 No

7. Do you discuss medicine prices with your physician? (during your visit or on the phone)

1 Never
2 Sometimes
3 Nearly always
4 Always

8. Has your physician ever forbidden you to substitute your medicine with an equivalent medicinal product at the pharmacy?

1 Yes
2 No → Go to question **10.**

9. Did your physician tell you why the medicine must not be substituted at the pharmacy?

1 No
2 Yes. Why? __________________________

____

THE FOLLOWING QUESTIONS CONCERN YOUR EXPERIENCES AT THE PHARMACY

10. What matters related to the <u>substitution of a prescription medicine</u> were you told about <u>during this pharmacy visit</u>? You may choose several options.

1 I have the possibility to substitute a medicine with an equivalent, but cheaper, one
2 I have the possibility to choose my medicine from among several alternatives
3 That there are price differences between interchangeable medicines
4 That there are differences in composition between interchangeable medicines (e.g. whether the product contains lactose)
5 That there are differences between interchangeable medicines in terms of packaging (e.g. container or blister)
6 That there are differences in the appearance of interchangeable medicines (e.g. tablet shape, colour)
7 Which pharmaceutical company has manufactured the medicine
8 About reimbursability of the medicine by the Social Insurance Institution (Kela)
9 About availability of interchangeable medicines (e.g. whether the medicine is available at the pharmacy or whether it must be ordered by the pharmacy)
10 If I wish, I may choose not to substitute my medicine with an equivalent, but cheaper, medicinal product
11 About other factors affecting the choice of the medicine; please specify___________________________

____

____

11. Did the dispenser/pharmacist offer you <u>the cheapest</u> of the interchangeable medicines <u>during this pharmacy visit</u>?

1 Yes
2 No → Go to question **13.**

12. Did you choose the cheapest medicinal product offered by the dispenser/pharmacist?

1 Yes
2 No

13. In your opinion, did you receive enough information about the substitution of prescription medicines <u>during this pharmacy visit</u>?

1 Yes
2 No. About which matters do you wish to have further information?______________________________

____

____

____

14. When you think about <u>this pharmacy visit as a whole</u>, would you say that it went well?

1 Yes
2 No. What problems did you have? ____

____

____

THE FOLLOWING QUESTIONS CONCERN YOUR EXPERIENCES AND OPINIONS ABOUT SUBSTITUTION OF MEDICINES

15. Have you ever substituted your prescription medicine with an equivalent medicinal product at the pharmacy?

1 Yes
2 No → Go to question **17.**

16. What were the main reasons for your decision to substitute your medicine with an equivalent medicinal product? You may choose several options.

1 I wanted to lower my medicine expenses
2 I was recommended to substitute my medicine at the pharmacy
3 My physician recommended me to substitute my medicine
4 The medicine prescribed by my physician was not available at the pharmacy
5 The medicine I had used before was not available at the pharmacy
6 I was dissatisfied with the medicine I had used before
7 Some other reason; please specify____

____

17. Have you ever chosen not to substitute your prescription medicine with a cheaper equivalent medicinal product offered by the dispenser?

1 Yes
2 No → Go to question **19.**

18. What were the main reasons for your decision not to substitute your medicine with a cheaper equivalent medicinal product? You may choose several options.

1 I did not receive enough information about different product alternatives
2 I thought difference in price of the interchangeable medicines was too small
3 I have used the medicine before and consider it a good one
4 The equivalent medicinal product I wanted was not available at the pharmacy
5 My medicines have often had to be switched before a suitable one was found
6 During my visit to the physician, we ended up choosing the alternative recommended by the physician
7 I fear that I will mix up my medicines if I substitute my medicine with an equivalent one
8 I'm concerned the equivalent medicinal product will not be as effective as the medicine I've used before
9 I'm concerned the equivalent medicinal product will not be as effective as the one prescribed by my physician
10 My medicines are subject to special reimbursement, so I would not have benefited from the saving
11 I do not pay my medicine expenses myself
12 Some other reason; please specify____

____

19. Where have you received information about the possibility to substitute a medicine with a cheaper equivalent medicinal product at the pharmacy? You may choose several options.

1 Nowhere
2 From a physician
3 From the pharmacy
4 From a relative/friend
5 From the media
6 From the website of the Social Insurance Institution
7 From the internet; from which sites?_________________________
8 Somewhere else, where? ___________________________________

20. If your physician has prescribed you a medicine which you can substitute with another, equivalent medicinal product at the pharmacy, which of the following factors matter when you are choosing a prescription medicine at the pharmacy? You may choose several options.

1 Familiarity of the medicine
2 Price of the medicine
3 Tablet/capsule colour
4 Tablet/capsule shape
5 Possibility to halve the tablet
6 Medicine pack (e.g. container vs. blister)
7 Excipients contained in the product (e.g. lactose)
8 The medicine is Finnish
9 Availability of the medicine
10 Manufacturer of the medicine / pharmaceutical company
11 Product name / brand / name of the medicine
12 Other – please specify
____
____

21. What do you think about the following statements? For each, circle the number of the option you consider appropriate.

	**Completely agree**	**Agree to some extent**	**Disagree to some extent**	**Completely disagree**	**Don't know**
I think that the possibility to substitute a medicine at a pharmacy with an equivalent, but cheaper, one is a good thing	1	2	3	4	5
I'm happy to substitute my medicine with a cheaper equivalent one at the pharmacy	1	2	3	4	5
I think that substitution of medicines is simple	1	2	3	4	5
I think that cheaper medicinal products are effective	1	2	3	4	5
At the pharmacy, I want to be informed of the differences in price between interchangeable medicinal products if the difference is less than 0.50 euros	1	2	3	4	5
I want to be offered the cheapest of the interchangeable medicinal products	1	2	3	4	5
I want my physician to discuss medicine prices with me	1	2	3	4	5
I think that cheaper medicinal products are safe	1	2	3	4	5

Here you can write your comments about this survey and your experiences of generic substitution. If necessary, you can continue e.g. on the back of the cover letter and return it with the questionnaire.

____
____
____
____
____

Thank you!
